# Comment on ‘A Causal Effect of Serum 25(OH)D Level on Appendicular Muscle Mass: Evidence From NHANES Data and Mendelian Randomization Analyses’ by Ren et al.

**DOI:** 10.1002/jcsm.13855

**Published:** 2025-06-04

**Authors:** Bo Zeng, Chaoqun Zhou, Jinhua Liu, Yongbo Zhu

**Affiliations:** ^1^ Department of Otolaryngology‐Head and Neck Surgery The First Affiliated Hospital of Yangtze University Jingzhou Hubei Province China; ^2^ Department of Oncology The First Affiliated Hospital of Yangtze University Jingzhou Hubei Province China

Serum 25‐hydroxyvitamin D [25(OH)D] may enhance skeletal muscle metabolism and increase muscle mass through activation of vitamin D receptor pathways, reduction of oxidative stress and mitigation of mitochondrial dysfunction and cellular senescence. However, due to inherent limitations associated with traditional observational studies, previous findings remain inconsistent. Mendelian randomization (MR), leveraging genetic variants as instrumental variables, has emerged as a pivotal approach for investigating potential causal relationships. In a recent integrated observational and MR study, Ren et al. identified a causal effect of genetically predicted serum 25(OH)D levels on appendicular muscle mass (AMM) in European populations [1]. Nevertheless, it is important to note that this causal inference—whether derived from univariable or multivariable analyses—may be an artefact of statistical bias, known as the ‘winner's curse’, attributable to the substantial sample overlap between exposure and outcome datasets.

In recent years, rapid advancements in genetic technologies and the extensive implementation of genome‐wide association studies (GWAS) have provided new opportunities for causal inference research. Numerous large‐scale databases and research consortia, such as the UK Biobank (UKB) and the Genetic Investigation of Anthropometric Traits (GIANT) Consortium, have made summary‐level GWAS data publicly accessible. This availability has enabled researchers to conduct two‐sample MR analyses across cohorts and platforms, integrating data from diverse sources. The primary advantages of this approach include a significant increase in sample size, thus enhancing statistical power, and the inclusion of populations from various ethnicities and backgrounds, thereby improving the generalizability and external validity of research findings.

However, these benefits also come with inherent risks. Given that large‐scale meta‐GWAS analyses tend to identify a greater number of genetic variants significantly associated with traits, meta‐analysis across multiple cohorts has become the dominant strategy in current GWAS research. Consequently, a frequent concern in GWAS‐based MR analyses is that the exposure and outcome data may originate from identical or substantially overlapping samples, potentially leading to biassed estimates. The study by Ren et al. exemplifies this issue, demonstrating sample overlap ranging from 8.93% to 98.65% between exposure and outcome datasets (Table 1). Specifically, in single‐sample analyses, serum 25(OH)D (*n =* 417 580) and AMM (*n =* 450 243) were both derived from the UKB, resulting in an overlap of up to 92.75%. When there is substantial sample overlap between datasets used for the exposure and outcome in MR studies, the estimated effect sizes of single nucleotide polymorphisms (SNPs) from the exposure GWAS can be inflated due to stochastic variability. These inflated estimates subsequently propagate into and amplify biases within the outcome GWAS. Additionally, overlapping samples violate the assumption of independence between exposure and outcome datasets, creating statistical dependencies that introduce bias into causal inference. Such violations undermine the validity of instrumental variable analyses and increase the likelihood of type I errors, a phenomenon commonly referred to as the ‘winner's curse’. [2] Evidence indicates that under scenarios involving complete sample overlap, causal estimates derived from inverse variance weighting (IVW) methods may be inflated by approximately 30%, with reported false‐positive rates ranging from 20% to 40% [3]. In the multivariable MR analysis, Ren et al. similarly failed to avoid the influence of the winner's curse. Specifically, the covariates adjusted for—including body mass index (BMI), average household income before tax, years of schooling, and physical activity—were all derived from the UKB cohort (*n =* 40 210–456 426). Consequently, these covariates shared substantial sample overlap with the outcome dataset, ranging from 8.93% to 98.65%. Apart from the previously mentioned impacts, this overlap also interferes with covariance estimation in the model, leading to inaccurate confounder adjustment. Such biases can obscure the true nature of observed effects, making it challenging for researchers to ascertain whether associations between the exposure and outcome are genuinely independent of confounding influences.

**TABLE 1:** Summary of sample overlap rate calculations.

Phenotype 1	Sample size	UKB samples	Phenotype 2	Sample size	UKB samples	Overlapping study	Sample overlap
Serum 25(OH)D	417 580	417 580	AMM	450 243	450 243	UKB	92.75% (417 580/450243)
Serum 25(OH)D	417 580	417 580	BMI	681 275	456 426	UKB	91.49% (417 580/456426)
Serum 25(OH)D	417 580	417 580	Average household income before tax	397 751	397 751	UKB	95.25% (397 751/417580)
Serum 25(OH)D	417 580	417 580	Years of schooling	766 345	442 183	UKB	94.44% (417 580/422183)
Serum 25(OH)D	417 580	417 580	Physical activity	78 007	40 210	UKB	9.63% (40 210/417580)
BMI	681 275	456 426	AMM	450 243	450 243	UKB	98.65% (450 243/456426)
Average household income before tax	397 751	397 751	AMM	450 244	450 244	UKB	88.34% (397 751/450243)
Years of schooling	766 345	442 183	AMM	450 245	450 245	UKB	98.20% (442 183/450243)
Physical activity	78 007	40 210	AMM	450 246	450 246	UKB	8.93% (40 210/450243)

In such situations, we primarily recommend the use of exposure and outcome datasets derived from distinct, nonoverlapping cohorts—such as those provided by the FinnGen Consortium or the Million Veteran Program (MVP)—to eliminate potential bias stemming from sample overlap at its source. When such overlaps are unavoidable, we suggest employing available online tools (https://sb452.shinyapps.io/overlap/) designed to assess the magnitude of bias and control for inflated type I error rates. Moreover, we advocate for the application of MRLap [3], an analytical approach grounded in the ‘spike‐and‐slab’ hypothesis of genetic architecture, wherein only a limited subset of SNPs substantially influences the exposure phenotype. MRLap leverages linkage disequilibrium score regression (LDSC) to accurately quantify both trait heritability and polygenicity. Based on conditional normal distribution theory, this method corrects joint biases caused by the winner's curse—namely, inflated estimates of SNP effects—and the inclusion of weak genetic instruments (characterized by low heritability). Furthermore, MRLap incorporates a dynamic assessment of the extent of sample overlap using the LDSC cross‐trait intercept term (λ’), enabling precise adjustment of biases induced by confounding factors in both magnitude and direction. Prior evaluations have demonstrated robust performance of MRLap, even in scenarios characterized by sample overlap as high as 95%.

In summary, while we commend the efforts of Ren et al. in investigating the causal association between serum 25(OH)D levels and AMM, rigorous quality control is essential to ensure accurate causal inference and prevent misapplication or misinterpretation of MR analysis. Future research should explore the potential nonlinear causal relationships of serum 25(OH)D levels utilizing individual‐level genetic data and extend analyses to more diverse populations, thereby enhancing the generalizability and applicability of the findings.

## Conflicts of Interest

The authors declare no conflicts of interest.
